# Molecular characteristics and immune microenvironment of gastrointestinal stromal tumours: targets for therapeutic strategies

**DOI:** 10.3389/fonc.2024.1405727

**Published:** 2024-07-12

**Authors:** Yang Yu, Mengdie Yu, Lijie Luo, Zijing Zhang, Haiping Zeng, Yan Chen, Zeyu Lin, Mengnan Chen, Wei Wang

**Affiliations:** ^1^ Department of Gastrointestinal Surgery, The First Affiliated Hospital of Guangzhou University of Traditional Chinese Medicine, Guangzhou, Guangdong, China; ^2^ Guangzhou KingMed Diagnostics Group Co., Ltd., Guangzhou, Guangdong, China; ^3^ Department of Thyroid and Breast Surgery, Baiyun Hospital, The First Affiliated Hospital of Guangzhou University of Traditional Chinese Medicine, Guangzhou, Guangdong, China

**Keywords:** gastrointestinal stromal tumours, immunotherapy, targeted therapy, molecular characteristics, immune microenvironment

## Abstract

Gastrointestinal stromal tumours (GISTs) are the most common mesenchymal tumours, arising mainly from the interstitial cells of Cajal (ICCs) of the gastrointestinal tract. As radiotherapy and chemotherapy are generally ineffective for GISTs, the current primary treatment is surgical resection. However, surgical resection is not choice for most patients. Therefore, new therapeutic strategies are urgently needed. Targeted therapy, represented by tyrosine kinase inhibitors (TKIs), and immunotherapy, represented by immune checkpoint inhibitor therapies and chimeric antigen receptor T-cell immunotherapy (CAR-T), offer new therapeutic options in GISTs and have shown promising treatment responses. In this review, we summarize the molecular classification and immune microenvironment of GISTs and discuss the corresponding targeted therapy and immunotherapy options. This updated knowledge may provide more options for future therapeutic strategies and applications in GISTs.

## Introduction

Gastrointestinal stromal tumours (GISTs) arise from gastrointestinal stromal cells, accounting for about 4% to 5% of gastrointestinal malignancies (1). The incidence of GISTs has been increasing worldwide over the years, reflecting in part improvements in diagnostic techniques and a clearer pathological classification (2). Over the past few decades, the treatment of GISTs has mainly relied on conventional therapies such as surgical resection and radiotherapy (3, 4). However, with in-depth research into the pathogenesis of GISTs, there have been significant breakthroughs in the treatment of GISTs, particularly in the areas of targeted therapy and immunotherapy (5, 6).

GISTs typically have mutations in the KIT proto-oncogene, receptor tyrosine kinase (*KIT*) or platelet-derived growth factor receptor alpha (*PDGFRA*) genes, leading to uncontrolled cell proliferation through abnormal signalling pathways, which in turn provide therapeutic opportunities to target these molecules (7, 8). Patients with *KIT* mutations usually respond well to targeted therapies such as imatinib (9). However, some patients may develop resistance after long-term treatment, which is one of the main challenges of targeted therapy.

In contrast to drug-targeted therapy, immunotherapy uses the body’s own immune system to attack cancer cells and includes immune checkpoint inhibitors such as programmed cell death 1 (PDCD1; also known as PD-1) and cytotoxic T-lymphocyte associated protein 4 (CTLA4) inhibitors (10). These inhibitors prevent cancer cells from evading the immune system, thereby increasing the immune system’s ability to kill cancer cells. Several preclinical studies have shown that immunotherapy can induce a positive therapeutic response in a subset of GIST patients (11, 12).

In this review, we summarize the molecular classification and immune microenvironment of GISTs. We also focus on targeted therapy and immunotherapy for GISTs. A comprehensive and updated knowledge of the characteristics and treatment strategies of GISTs may provide more appropriate options for subsequent therapeutic choices and research.

## Molecular classification of GISTs

**Figure 1:**
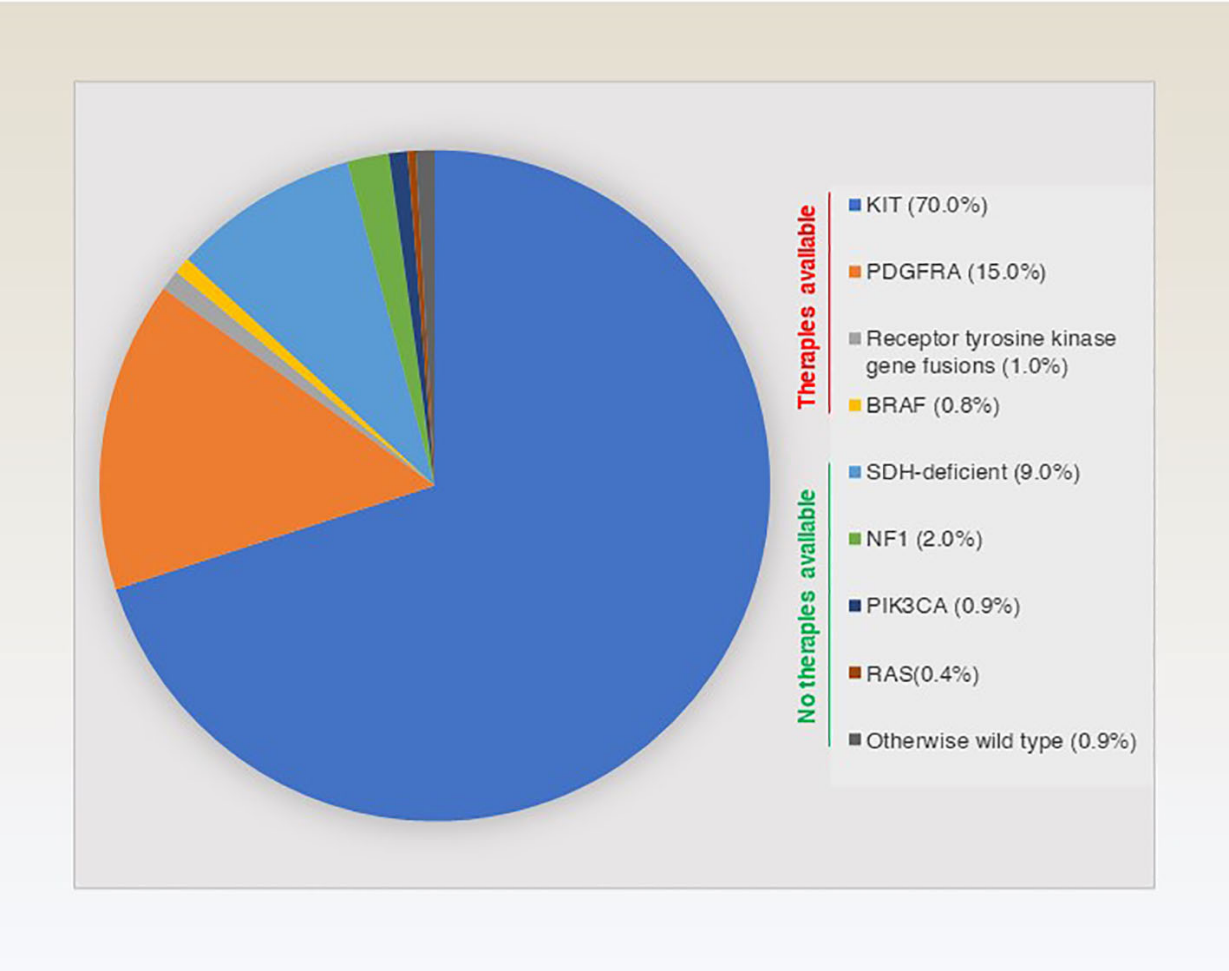
Overview and approximate percentage of molecular typing of genes driving GISTs.

**Figure 2:**
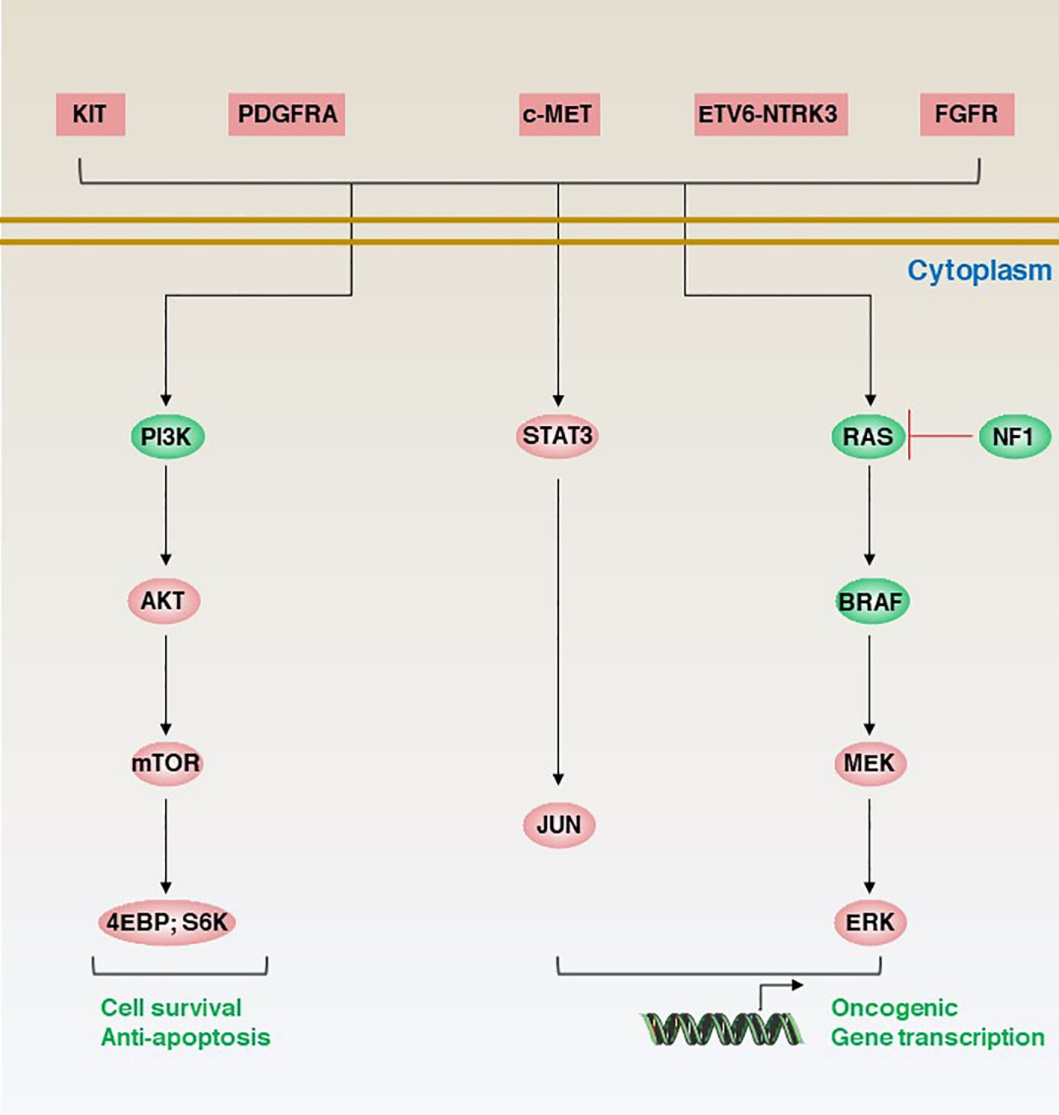
Genetic characteristics and molecular classification of induced GISTs.

**Figure 3:**
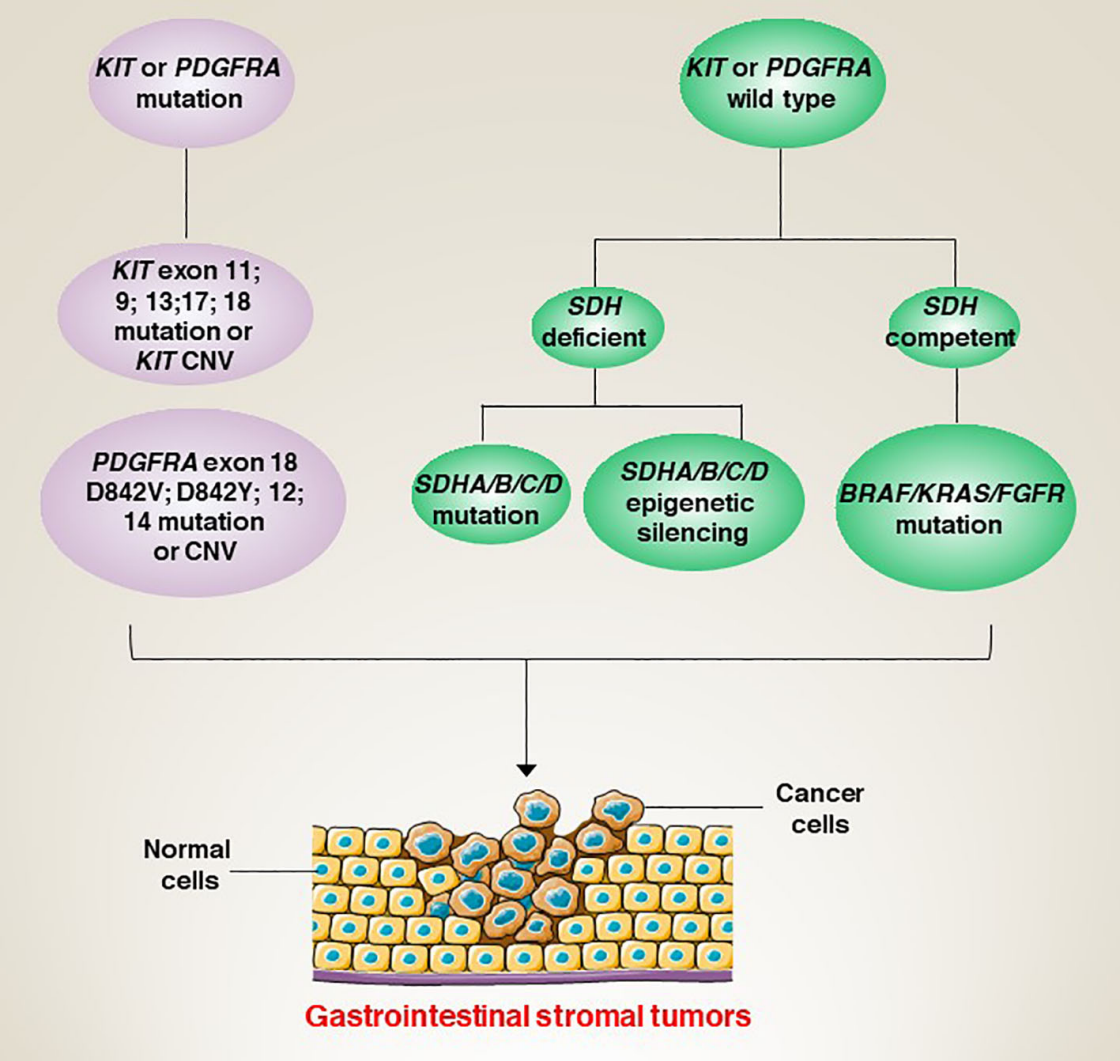
Aberrant genetic alterations in GISTs activate multiple signalling cascades thereby preventing apoptosis and promoting cell survival and proliferation.

The molecular classification of GISTs is mainly based on the presence of specific mutations in their tumour cells (**Figure 1**), mainly including *KIT*, *PDGFRA* and succinate dehydrogenase complex iron sulfur (*SDH*) (**Figure 2**), which result in the structural activation of specific signalling pathways in a ligand-independent manner, driving the onset and progression of GISTs (**Figure 3**).

### KIT

The *KIT* gene, located on chromosome 4q12, is a proto-oncogene that encodes a type III membrane-penetrating receptor tyrosine kinase protein (13). Under normal physiological conditions, KIT encoded proteins act as cell membrane surface receptors to regulate cell growth and differentiation and are present in a variety of cell types, including embryonic stem cells, haematopoietic stem cells, gastrointestinal stromal cells (interstitial cells of Cajal) and germ cells (14–18). In particular, KIT proteins play a key role in the regulation of gastrointestinal motility and muscle contraction (19). Thus, *KIT* mutations are a key driver of GISTs.

Depending on the location and type of mutation, *KIT* gene mutations can be divided into several subtypes. The most common type of mutation occurs in the juxtamembrane domain of the *KIT* gene, with *KIT* exon 11 mutations accounting for approximately 50-70% of GIST cases. *KIT* exon 11 mutations usually involve a deletion, insertion or point mutation in the region of the KIT protein enhancer, resulting in persistent activation of KIT protein kinase activity (20, 21). However, *KIT* exon 11 mutations are also usually associated with a better prognosis. For example, sequencing of tumour DNA from 104 GIST patients showed a significantly better trend for disease-free survival (DFS) in patients with exon 11 repeat mutations and point mutations compared to other mutations (e.g., deletions, insertions) (22). However, another study showed the opposite conclusion, which may indicate that tumour heterogeneity and the specific type of mutation are critical for prognosis (23). Another common mutant isoform of the *KIT* gene is *KIT* exon 9, which accounts for about 10-20% of cases. These mutations result in insertional mutations in the kinase region of the KIT protein, which in turn allows for sustained activation of the kinase activity of the KIT protein (24). In contrast, GISTs with *KIT* exon 9 mutations tend to have a higher tumour burden and a poorer prognosis (25).

In addition, there are some rare mutant subtypes of the *KIT* gene, such as *KIT* exon 13, exon 17 and exon 18 mutations (8, 26, 27). Mutations in each subtype may lead to different clinical features and prognosis of GIST. While *KIT* gene mutation-dependent aberrant signalling mainly involves mitogen-activated protein kinase (MAPK) and AKT serine/threonine kinase (AKT) (5, 28). In addition to mutations, copy number variation (CNV) in the *KIT* gene is an important genetic abnormality in the development of GISTs (29).

### PDGFRA

PDGFRA TKR is homologous to KIT TKR (30). Similarly, under normal conditions, PDGFRA signalling maintains a normal balance between cell growth and apoptosis through ligand binding and kinase activity (31, 32). However, in GISTs, mutations result in sustained activation of the kinase activity of these receptors. *PDGFRA* mutations account for approximately 10-15% of GISTs (33). Their mutations mainly affect its kinase structural domain and the most common mutation site is site 842 of exon 18, including point mutations (e.g., D842V, D842Y), deletion and insertion mutations (34). Among these, *PDGFRA* D842V mutations are the most common, accounting for approximately 70-80% of *PDGFRA* mutant GISTs (34, 35).

In particular, *PDGFRA*-mutant GISTs tend to have some unique clinical features and prognosis. First, *PDGFRA*-mutant GISTs are often found in the stomach, particularly in the gastric antrum and pylorus (36). Second, *PDGFRA*-mutant GISTs often have lower histological grades and lower proliferation indices, factors associated with a better prognosis (37, 38). In addition, *PDGFRA*-mutant GISTs tend to have smaller tumour sizes, a lower incidence of metastases and longer survival (39). GISTs with *PDGFRA* D842V mutations have a better prognosis but are relatively insensitive to conventional KIT inhibitors (e.g., imatinib) and therefore require higher doses of the drug to achieve an effective therapeutic response. For example, a retrospective cohort study of GIST patients with *PDGFRA* exon 18 mutation showed that 16 GIST patients with D842V mutation received imatinib treatment, and only 2 patients had partial response, indicating that imatinib is not generally applicable to patients with this mutation (40). On the other hand, GISTs with other types of *PDGFRA* exon 18 mutations (e.g., non-D842V mutations) are usually sensitive to therapeutic agents such as imatinib, have a relatively better prognosis and require lower doses of drugs for maintenance therapy (40). Similar to KIT, CNV (gene dose changes caused by chromosome gain and loss) in the *PDGFR*A gene is associated with the prognosis of GISTs (41, 42). In addition, the development of GISTs is closely linked to the interaction of other cell types in the tumour microenvironment, such as tumour-associated mesenchymal stromal cells and immune cells (43, 44).

### SDH

In addition to *KIT*/*PDGFRA* mutations. There is also a small percentage of GISTs (~5-7.5%) whose pathogenesis is closely linked to a deletion of the succinate dehydrogenase (SDH) complex (45). SDH is a mitochondrial enzyme that plays an important role in energy metabolism and oxidative stress in cells (46). Therefore, *SDH* mutations cause mitochondrial dysfunction, leading to abnormal intracellular energy metabolism and increased oxidative stress, which promotes the growth and division of tumour cells (47, 48). *SDH* mutations can be classified into four isoforms, namely SDHA, SDHB, SDHC and SDHD (49). Notably, *SDH*-mutant GISTs tends to occur in younger patients, and the tumours are smaller and less malignant, suggesting that it may have a better prognosis (50). However, *SDH*-mutant GISTs respond poorly to treatment with conventional targeted agents (such as imatinib) and are relatively sensitive to other treatments, such as chemotherapy (51). In a study based on the molecular and metabolic profiles of a patient-derived *SDH*-mutant (mSDH) GIST model, temozolomide induced tumour DNA damage and apoptosis in the mSDH GIST model. Importantly, five patients with *SDH*-mutant GISTs who received temozolomide showed an objective remission rate of 40% and a disease control rate of 100%, suggesting that temozolomide is a promising therapeutic approach (52).

In addition, there is a small percentage of GIST patients who do not have mutations and these patients are referred to as ‘wild-type’ GISTs (53). Therefore, the development of multidisciplinary (such as combined genetic testing and tissue dynamic imaging techniques) and accurate assays to determine the genomic background of different patients is essential for precision therapy.

### GIST targeted drug therapy

**Figure 4:**
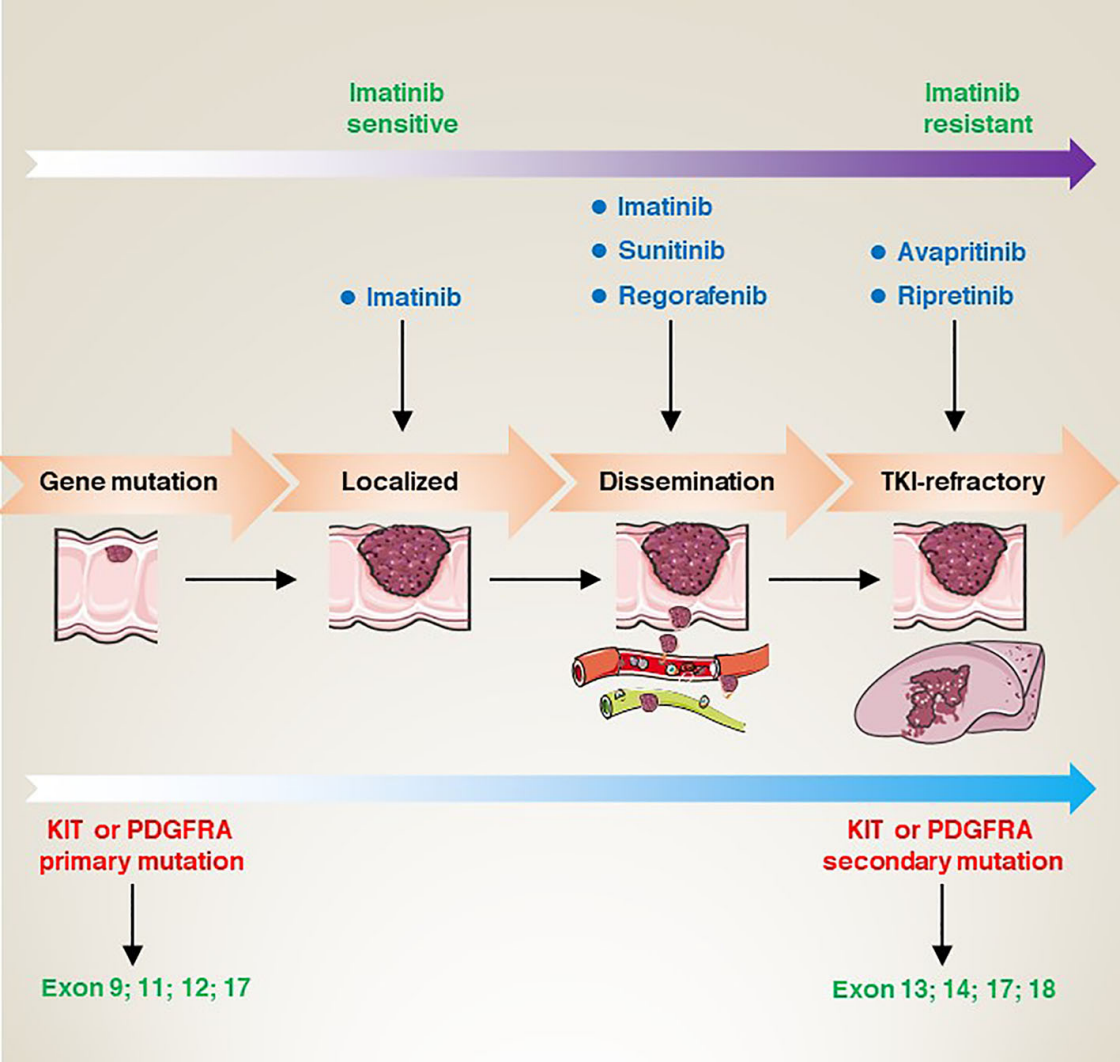
From gene mutation to clinical refractory GISTs, the corresponding site mutation type, the sensitivity changes of imatinib, and the targeted drugs at each stage.

Targeted therapies have revolutionized the treatment strategies for GISTs over the last few decades. Many drugs have been approved by the U.S Food and Drug Administration (FDA) for the treatment of GISTs, and these drugs mainly target mutant *KIT* and *PDGFRA*. In this section, we mainly focus on targeted therapy for KIT or PDGFRA mutations (**Figure 4**).

### Targeting *KIT* or *PDGFRA*

Most GIST patients have acquired mutations in the *KIT* or *PDGFRA* genes. Typically, these patients show a favourable response to TKI-targeted therapy. Historically, GISTs were thought to be smooth muscle or neurogenic tumours. However, in 1998, researchers first identified the presence of mutations in the *KIT* gene in patients with GISTs (7). In subsequent studies, researchers also identified functionally acquired mutations in the *PDGFRA* gene, which are also strongly associated with the development of GISTs (54). These findings have led to a surge in the development of drugs that target *KIT* and *PDGFRA* mutations. Imatinib (IM) became the first FDA-approved first-line treatment for unresectable and metastatic GISTs in 2002 (55). Imatinib selectively inhibits the activity of KIT receptors. Preliminary clinical trial results show that imatinib has a significant therapeutic effect on *KIT* mutation-positive GISTs patients (55). For *KIT*/*PDGFRA* mutant GIST with a high risk of disease recurrence, 3 years of 400 mg/d imatinib adjuvant therapy may prolong relapse-free survival. In addition, for GIST patients at high risk of relapse, the duration of adjuvant therapy with IM (800 mg/d) (3 years) resulted in a longer progression-free survival (the hazard ratio 0.39; 95% CI; 0.22-0.71, *P*=0.0013) in patients with *KIT* exon 9 GISTs (56).

However, although imatinib showed good efficacy in most patients, some patients developed resistance or no response. For this reason, after relentless efforts and long-term preclinical studies, sunitinib (SU) was developed as a second-generation targeted agent for the treatment of drug-resistant GISTs patients (57, 58). A randomized controlled trial on the efficacy and safety of sunitinib in patients with advanced gastrointestinal mesenchymal stromal tumours who failed imatinib therapy was evaluated in 2006. The results showed that in 312 patients randomized 2:1 to receive sunitinib (n = 207) or placebo (n = 105), the median time to tumour progression was significantly delayed in patients in the sunitinib group (27.3 weeks) compared to the median time to tumour progression in the control group (6.4 weeks) (57). Subsequently, the FDA re-approved regorafenib as a third-line drug for the clinical treatment of advanced GISTs patients who failed in both IM and SU treatment (59).

In addition, due to the diversity of *KIT* and *PDGFRA* mutations in GISTs patients, therapeutic strategies need to be individualized according to the type of mutation (60, 61). For example, *PDGFRA D842V* mutant GISTs are usually resistant to IM (62). Therefore, ripretinib has been developed as an inhibitor targeting the *PDGFRA* D842V mutation (63–65). Moreover, several other targeted agents have been used to treat patients with IM-resistant GISTs, such as avapritinib (BLU-285) (66).

As research into GISTs has deepened, IM as the preferred drug has shown good responses in most patients with *KIT* or *PDGFRA* mutations, but not complete cures. Patients still have a high chance of developing resistance during follow-up, although SU or regorafenib are approved for the treatment of advanced or resistant GISTs. Thus, drug resistance remains a challenge. To address this problem, exploring different new inhibitors or by combining different drugs and individualized treatment regimens can maximize patient outcomes (67).

### The immune microenvironment of GIST

**Figure 5:**
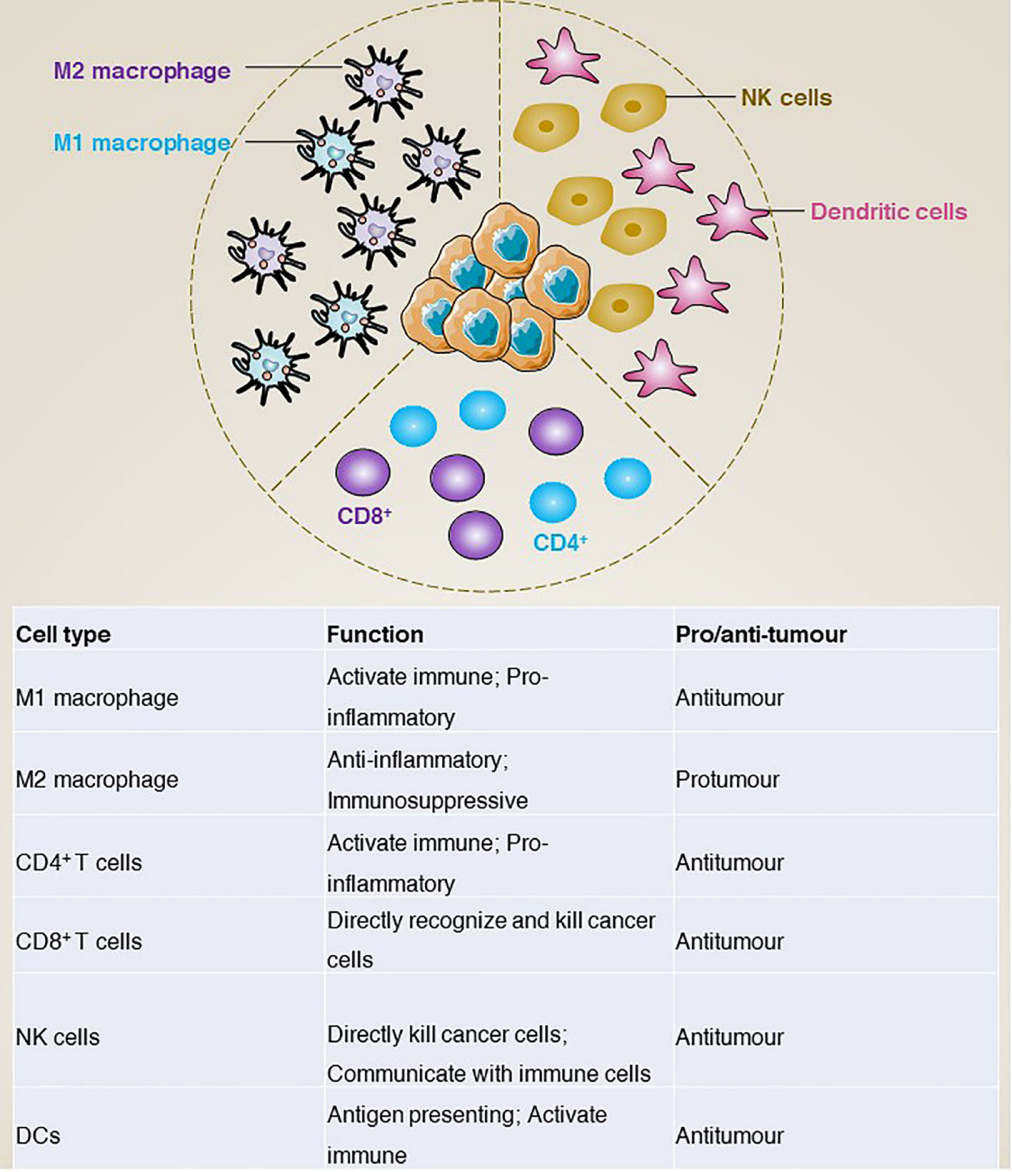
The main immune infiltrating cells and corresponding functions of GISTs microenvironment.

In addition to gene mutations, the tumour immune microenvironment is increasingly considered to play a key role in tumours. In GISTs, tumour-associated macrophages (TAM) and CD3^+^ tumour-infiltrating lymphocytes (TIL) are the most common immune cells. In addition, natural killer (NK) cells, dendritic cells (DCs), and natural killer T (NKT) cells are also included. In this section, we mainly review how these immune cells are involved in the development of GISTs (**Figure 5**).

### Tumour-associated macrophages

Macrophages are an important class of cells in the immune system, mainly involved in non-specific and innate immune responses (68). In the tumour environment, tumour-associated macrophages can be classified into two main groups according to their surface molecules, origin and functional characteristics: M1 type macrophages and M2 type macrophages (69). Functionally, M1 type macrophages activate the immune response mainly through the production of pro-inflammatory cytokines (e.g., tumour necrosis factor-α [TNFα], interleukin-1β [IL-1β], interleukin-6 [IL-6]) and inhibit tumour growth and spread by phagocytosis and killing of tumour cells (70–73). In addition, M1 macrophages promote the activation of T cells, thereby enhancing specific immune responses (74, 75). In contrast, M2 macrophages produce anti-inflammatory cytokines (e.g., IL-10, transforming growth factor-β [TGFβ]) to modulate the immune response and inhibit T-cell activation and killing (76, 77), thereby promoting tumour evasion of immune surveillance (78). For example, cancer-associated fibroblasts activated by cancer cells release IL-6 and granulocyte-macrophage colony-stimulating factor (GM-CSF) to induce monocytes to differentiate into M2-like TAMs, activate the immunosuppressive tumour microenvironment and promote GISTs metastasis (79). However, although macrophage polarization in the tumour microenvironment of primary untreated GISTs is controversial, M2 macrophages predominate in metastatic or treated GISTs. For example, imatinib treatment of GISTs induces apoptosis in tumour cells, which further induces TAM M2-like polarization via CCAAT enhancer binding protein beta (CEBPB) (80).

Together, TAMs regulate the development and progression of GISTs through various mechanisms, including promoting tumour growth and dissemination and suppressing the immune response.

### Tumour-infiltrating lymphocytes

Tumour-infiltrating T lymphocytes are a class of immune cells that have anti-tumour capacity and are present in tumour tissue (81). According to the phenotypic characteristics, they can be divided into CD4^+^ and CD8^+^ T cells (82). CD4^+^ T cells activate other immune cells and regulate the immune response mainly by releasing cytokines (83). In contrast, CD8^+^ T cells can directly recognize and kill cancer cells (84). Depending on their function in the tumour microenvironment, tumour-infiltrating T lymphocytes can be further classified into effector T cells (85), regulatory T cells and memory T cells (86), which play the functions of direct tumour killing, suppression of the immune response and recognition of resistance to tumours, respectively.

The presence of abundant tumour-infiltrating immune cells in GISTs has been implicated in the regulation of GISTs through mechanisms such as direct killing of GIST cells, stimulation of immune responses and modulation of immunosuppression. For example, imatinib treatment decreased the frequency of effector CD8^+^ T cells and increased the frequency of naive CD8^+^ T cells. Mechanistically, this was mainly due to the production of tumour chemokines and the reduction of intracellular phosphatidylinositol-4,5-bisphosphate 3-kinase catalytic (PI3K) signalling in CD8^+^ T cells. In contrast, IL15 super-antagonist (IL15SA) in combination with imatinib significantly restored intratumoural effector CD8^+^ T cell function and intracellular PI3K signalling in CD8^+^ T cells, thereby enhancing tumour killing (87). Similarly, in spontaneous GISTs, imatinib inhibits the expression of the immunosuppressive enzyme indoleamine 2,3-dioxygenase (IDO), which activates CD8^+^ T cells in the tumour and induces apoptosis of regulatory T cells (T(reg) cells), thereby enhancing the immunotherapeutic effect in a mouse model (88). However, despite the presence of abundant CD8^+^ infiltration in GISTs, the effect of immune checkpoint inhibition in combination with TKI-targeted therapy in patients with advanced GISTs is rather limited (12).

Together, differences in response to imatinib between patients with early and advanced GIST suggest that there are also different populations of immune cells that play an immunomodulatory role.

### Natural killer cells

Tumour-infiltrating natural killer (NK) cells are the first line of defence in the fight against infection and tumour (89). NK cells not only kill tumour cells directly by releasing cytotoxins and cytokines, such as perforin and interferon gamma (90, 91), but also recognize and interact with other immune cells, such as dendritic cells and macrophages, to modulate the GISTs immune response (92, 93). The molecular phenotype of tumour-infiltrating NK cells is closely linked to their function, and their surface molecules include activating and inhibitory receptors such as KIR (killer cell immunoglobulin-like receptor) and killer cell lectin like receptor C1 (KLRC1; also known as NKG2A) -mediated inhibitory signalling (94, 95). These receptors can interact with ligands on the surface of tumour cells to inhibit signalling pathways and prevent NK cell activation. In contrast, killer cell lectin like receptor K1 (KLRK1; also known as NKG2D) (96), CD226 molecule (CD226; also known as DNAM-1) and natural cytotoxicity triggering receptor 1 (NCR1; also known as NKp46) can bind to ligands on the tumour surface and activate NK cells, causing them to release cytotoxins (97, 98). For example, in addition to targeting KIT and PDGFRA, imatinib has also been shown to act on host dendritic cells to enhance anti-tumour effects *in vivo* by promoting NK cell activation (99).

In addition, NK cell infiltration is strongly associated with the prognosis of GISTs. For example, high expression of the immunosuppressive receptor for NK cells, natural cytotoxicity triggering receptor 3 (NCR3; also known as NKp30), in GISTs is negatively associated with patient survival. Mechanistically, NKp30 expression leads to a reduction in tumour necrosis factor-α (TNF-α) and CD107a release, as well as defects in interferon-γ (IFN-γ) and interleukin-12 (IL-12) secretion in the NK-DC cross-talk, which can be restored by blocking IL-10 (100). Similarly, the ligand for NKp30, natural killer cell cytotoxicity receptor 3 ligand 1 (NCR3LG1; also known as B7-H6), whose soluble form, sB7-H6, was negatively associated with DFS and prognosis in metastatic GISTs (101). While the production of IFN-γ by NK cells in patients treated with imatinib can also be considered an independent predictor of long-term survival in IM-treated advanced GIST (102). In addition, cytokine-secreting CD56 (NCAM1) NK cells were found to be enriched in tumour lesions of imatinib-treated patients and independently predicted progression-free survival (PFS) (103).

In summary, tumour-infiltrating NK cells are involved in the development and prognosis of GISTs through a variety of functions, including direct recognition and killing of tumour cells, production of cytokines and regulation of the immune response. However, the interactions and mechanisms between GISTs and tumour-infiltrating NK cells need to be further investigated in order to develop new immunotherapeutic strategies and improve the outcome of GISTs patients.

## Immunotherapy for GIST

**Figure 6:**
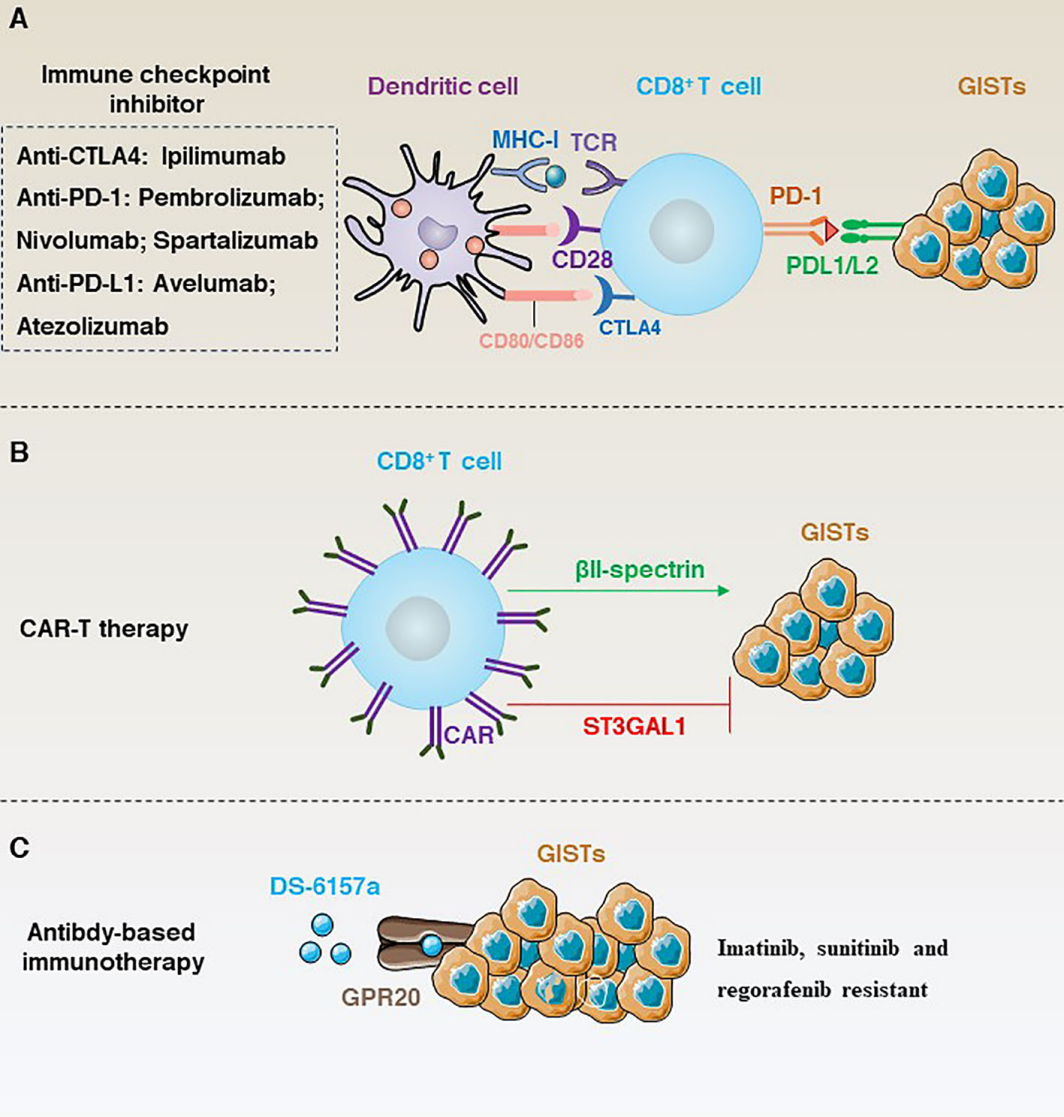
**(A)** Immune checkpoint inhibition-dependent anti-GISTs immunotherapy and corresponding inhibitors. **(B)** ST3GAL1 inhibits the localization of CAR-T cells to tumour sites, whereas βII-spectrin enhances the localization of CAR-T cells to tumour sites. **(C)** DS-6157a targets non-tyrosine kinases GPR20 to inhibit the immunotherapy of drug-resistant GISTs.

In the course of exploring the path of targeting GISTs, from the efficacy of first-line drugs to drug resistance and then continuous iterative updates. However, the efficacy in prolonging the PFS of patients is quite limited, and the targeted treatment of GISTs is in a therapeutic bottleneck. Therefore, immunotherapy, as a new generation of therapies, has begun to have a profound impact on the clinical management of solid tumours. In this section, we focus on immunotherapy based on immune checkpoint inhibition, CAR-T cells and antibody-dependent immunotherapy (**Figure 6**).

### Targeting immune checkpoint

Immune checkpoints are a class of inhibitory receptors and associated signals used by the immune system to maintain the body’s immune homeostasis, preventing damage to the body’s own tissues and maintaining tolerance to self-antigens (104). However, tumour cells can also use this mechanism to evade immune surveillance. Therefore, immune checkpoint inhibition can activate the immune system by blocking inhibitory signals at immune checkpoints to enhance its ability to attack tumour cells. Currently, the main immune checkpoints targeted in clinical trials include CTLA-4 (105), PD-1/PD-L1 (106), lymphocyte activating 3 (LAG3) (107), galectin-9 (LGALS9) and hepatitis a virus cellular receptor 2 (HAVCR2; also known as TIM3) (108, 109).

Typically, cancers that respond well to targeted immune checkpoints are characterised by an immune microenvironment infiltrated with high numbers of immune cells and pro-inflammatory cytokines such as CD4^+^ T cells, CD8^+^ T cells, interferon (IFN), IL12, IL2, IL23 and TNF-alpha. Cancer cells with this characteristic are called “hot tumours” (110). In contrast, “cold tumours” are characterized by low TIL counts, low PD-L1 expression and infiltration of large numbers of immunosuppressive cells and lack sensitivity to immunotherapy (110). GISTs are often considered to be “cold tumours” (111). Therefore, the use of immune checkpoint therapy in GISTs is relatively limited. However, some studies have shown that immune checkpoint inhibitors may have some benefit in a subset of GISTs patients (**Figure 6A**). For example, PD-1, LAG3 and TIM3 are significantly upregulated in tumour-infiltrating T cells compared to normal T cells. Importantly, In *Kit^V558Δ/+^* mice blockade of PD-1 and PD-L1 alone did not elicit an immune response, whereas the use of imatinib not only targeted KIT, but also inhibited IFNγ-induced upregulation of PD-L1 by inhibiting signal transducer and activator of transcription 1 (STAT1), significantly improving T cell effector function (112).

CD40 is a transmembrane protein that is widely expressed in DCs, B cells and monocytes, and is activated by binding to its ligand CD40LG (also known as CD154), mediating a variety of immune and inflammatory responses that limit therapeutic efficacy in GISTs (113). For example, CD40 is significantly inhibited in tumour-associated macrophages and tumour cells in GIST patients treated with imatinib. However, CD40 inhibition alone had no direct effect on human GISTs, whereas imatinib in combination with a CD40 antagonist significantly inhibited GISTs via the nuclear factor kappa B subunit 1 (NFKB1) pathway in mice carrying a mutation in *Kit* exon 11 (114).

In addition, the rate-limiting enzyme of human tryptophan metabolism, indoleamine 2,3-dioxygenase (IDO), has been identified as an immune checkpoint. For example, inhibition of IDO expression in tumour cells by imatinib activates CD8^+^ T cells in the tumour and induces T(reg) cell apoptosis, which, in combination with immunotherapy, significantly enhances the therapeutic effect of imatinib in mice (88). Similarly, a phase 2 clinical trial (NCT02406781) showed that PD-1 inhibition alone was not sufficient to control tumour growth and that a combination of inhibition of the colony stimulating factor 1 receptor and the IDO pathway resulted in more effective tumour control (12).

Together, immunotherapy for GISTs still faces many challenges and requires a better understanding of immune escape mechanisms and biomarkers that predict response to immunotherapy. In addition, individualization of therapy is an important challenge as pathological features and gene expression may differ from patient to patient. Therefore, in-depth analysis of patient data and integration of clinical trial results may improve maximum therapeutic efficacy.

### Chimeric antigen receptor T-cell therapy

Chimeric antigen receptor T-cell (CAR-T) is a treatment that modifies a patient’s own T cells so that they can recognize and attack cancer cells (115). The first generation of CAR mainly consisted of structural combinations of CD4 and CD3 (116), but due to the lack of potency a second generation of CAR with co-stimulatory structural domains such as CD28 or TNF receptor superfamily member 9 (TNFRSF9; also known as 4-1BB) is developed (117). CAR-T revolutionized the treatment of haematological cancers in 2010 when Professor Carl June took CAR-T cell therapy into human clinical trials and successfully cured leukaemia patients (116).

Unlike some conventional immunotherapies, such as TIL, CAR-T is not dependent on MHC antigens, and protein-like or lipid antigens specific to the tumour surface can be used as CAR-T targets (118). Given the remarkable effect of CAR-T in the treatment of haematological tumours. Many studies have tried CAR-T in GISTs using KIT/PDGFRA as a target to overcome resistance (**Figure 6B**). For example, Katz’s team constructed a novel anti-KIT chimeric immune receptors (CIR) with mouse and human T cells and effectively reduced tumour growth rates in a GIST mouse model (119). However, advancing *in vitro* trials to clinical trials in humans is a long process. To date, CAR-T clinical results in solid tumours have been unsatisfactory. Therefore, research into the negative regulatory mechanisms that modulate CAR-T may provide a solution. For example, a recent study has shown that ST3 β-galactoside α-2,3-glycosyltransferase 1 (ST3GAL1) is a negative regulator of cancer-specific migration of CAR-T cells. ST3GAL1-mediated glycosylation induces spontaneous non-specific tissue chelation of T cells by altering endocytosis of integrin subunit alpha l (ITGAL; also known as LFA-1). In contrast, βII-spectrin, a cytoskeletal molecule expressed on CAR-T cells, enhances the tumour specificity of CAR-T cells to localize to the tumour site and is able to reverse ST3GAL1-mediated non-specific T-cell migration, thereby inhibiting tumour growth in mice (6).

Together, CAR-T may be a potential treatment to overcome GISTs resistance. However, current research is still in preclinical studies, and the exploration of more specific CAR-T targets may lead to further advances in clinical trials.

### Antibody-based immunotherapy

TKIs are currently the only approved drugs for the treatment of GISTs, but GISTs are highly susceptible to secondary resistance. In contrast, monoclonal antibody therapy has shown promising therapeutic results in many tumours. Therefore, the development of new monoclonal antibodies targeting KIT, PDGFRA, CD40 and somatostatin receptor type 2 (SSTR2) is worth exploring. For example, a phase II clinical trial analysed the efficacy of nituzumab (N) or nituzumab + ibritumomab (N+I) in 36 patients with refractory GISTs. Results showed that benign responses to therapy and long-term disease control were observed with both N and N+I, but no new safety signals were observed (120). Specifically, the majority of patients treated with N or N+I showed some improvement in median progression-free survival (PFS) and clinical benefit rate (CBR) compared to imatinib treatment.

In addition, targeting non-tyrosine kinases is another important line of research for the treatment of GIST resistance (**Figure 6C**). For example, G protein-coupled receptor 20 (GPR20) has been identified as an important non-tyrosine kinase that is selectively expressed in GISTs. The anti-GPR20 antibody-drug conjugate DS-6157a showed GPR20 expression-dependent anti-tumour activity in a variety of resistant lines (e.g., imatinib, sunitinib and regorafenib) (121). These results suggest that the exploration of GIST-selective non-tyrosine kinases as therapeutic targets is a potential therapeutic option.

## Discussion

GISTs are rare but challenging malignant tumours. Traditional treatments include surgical resection, radiotherapy and chemotherapy (122). However, in recent years, targeted therapy and immunotherapy, as emerging therapeutic strategies, have made some important advances in the treatment of GIST (123–126). First, targeted therapy is an important component of GIST treatment. Some targeted drugs, such as imatinib, sunitinib and regorafenib, block cancer cell proliferation and survival by inhibiting aberrant signal activation mediated by *KIT* or *PDGFRA* mutations. These drugs have shown significant efficacy in some GIST patients and are better tolerated than conventional chemotherapy. However, a significant proportion of patients are prone to secondary resistance, which has led to alternative regimens using immunotherapy as a tool. Immune checkpoint inhibitors are one of the most successful immunotherapeutic approaches available and have achieved significant clinical results in a variety of tumours (127).

However, studies in GIST are relatively limited. First, tumour adaptation to KIT/PDGFRA inhibition likely leads to apoptosis evasion and GISTs survival through two intertwined events. Second, GISTs are primarily composed of macrophages and T cells, followed by NK cells and B cells, and it is unclear what role the distribution of other immune cell subtypes plays in the clinical prognosis, evolution and immune evasion of GIST patients. Third, infiltration of M2 macrophages and Treg cells (12, 128), high expression of IDO on GIST cells and immunosuppressive receptors on NK cells, and defective expression of MHC-I on APC. Although CD8^+^ T cells were enriched in GISTs (111), the proportion of CD8^+^ T/Treg cells were relatively low. In addition, NK cells were found to be negative for CD69. These factors contribute to the formation of an immunosuppressive microenvironment, which may be responsible for the avoidance of immunotherapy in GISTs. Therefore, the development of a novel therapeutic modality for tumour treatment is needed, and the induction of non-apoptotic-dependent cell death in cancer cells is a promising modality, such as alkaliptosis, cuproptosis and ferroptosis (129–134). Finally, high Ki67 expression is an independent predictor of aggressiveness, malignant potential and poor survival prognosis in GISTs, whereas short-term administration of imatinib activates CD8^+^ T cells, DC cells and NK cells and inhibits Treg cells, thereby enhancing the host’s anti-tumour immune response. Whereas imatinib was used in the perioperative period and Ki67 index was assessed to improve surgical outcomes (135, 136).

This is despite the fact that at least five currently marketed drugs have resulted in improved prognosis for patients with GISTs. However, the clinical benefits of approved second-, third-, and fourth-line drugs are often unsatisfactory for drug-resistant patients. NB003 is a potent and selective small-molecule tyrosine kinase inhibitor targeting *KIT/PDGFRA*, which is designed to inhibit a broad spectrum of primary and acquired imatinib-resistant mutations in *KIT/PDGFRA* (137). Similarly, IDRX-42 has been shown to have significant effects on GISTs with *KIT* exon 13 mutations (138). However, it should be noted that there are differences in the efficacy of different drugs for different genotypes, and how to effectively differentiate between genotypes can provide the best treatment options for patients with advanced GISTs. And olverembatinib has significant efficacy in the treatment of TKI-resistant *SDH*-deficient GIST patients, filling the gap in the treatment of *SDH*-deficient GISTs (139).

In conclusion, the combination strategy of targeted therapy and immunotherapy is worth exploring to further improve the therapeutic efficacy of GISTs (16, 132). The identification of selectively expressed GISTs targets may further improve the response rate and durability of treatment. In addition, research into appropriate biomarkers for early detection is urgently needed to reduce the prevalence of GISTs.

## Author contributions

YY: Writing – original draft, Writing – review & editing. MY: Writing – review & editing. LL: Writing – review & editing. ZZ: Writing – review & editing. HZ: Writing – review & editing. YC: Writing – review & editing. ZL: Writing – review & editing. MC: Writing – review & editing. WW: Writing – original draft, Writing – review & editing.
